# Abnormal expression of HSP70 may contribute to PCOS pathology

**DOI:** 10.1186/s13048-019-0548-7

**Published:** 2019-08-09

**Authors:** Gengxiang Wu, Xue Hu, Jinli Ding, Jing Yang

**Affiliations:** 1 Reproductive Medical Centre, Renmin Hospital of Wuhan University, Wuhan, 430060 People’s Republic of China; 2 Hubei Clinical Research Center for Assisted Reproductive Technology and Embryonic Development, Wuhan, 430060 People’s Republic of China

**Keywords:** Polycystic ovary syndrome, Heat shock protein 70, Inflammatory factor, Testosterone

## Abstract

**Background:**

The mechanism of the pathological change of polycystic ovary syndrome (PCOS) is still unclear. Previous studies have shown that PCOS is a chronic nonspecific low-grade inflammatory condition, and that heat shock protein (HSP)70 has a potent anti-inflammatory property. So the aim of this study is to investigate the correlation between HSP70 and the hormones and inflammatory factors and to find out the role of HSP70 in the pathogenesis of PCOS.

**Methods:**

Twenty female Sprague–Dawley (SD) rats (aged 23 days and weighted 80-90 g) were randomly divided into two groups (*n* = 10 per group), PCOS group and control group. PCOS group were subcutaneously injected with 6 mg/100 g dehydro-epiandrosterone (DHEA) for 20 consecutive days, the control group were subcutaneously injected with a solvent of equivalent amount. All the samples were collected in the morning fasting state, 12 h after the last administration. Histological examinations of ovarian tissues were analyzed. Hormone levels and inflammatory factors levels were measured by enzyme-linked immunosorbent assay (ELISA) kits.

**Results:**

Serum concentrations of testosterone (T) and luteinizing hormone (LH) were significantly higher in the PCOS group than the control group (*P* < 0.001), but the concentrations of estradiol (E_2_), follicle stimulating hormone (FSH) and insulin didn’t show significant difference between these two groups. All the concentrations of inflammatory factors including C-reactive protein (CRP), interleukin (IL)-6, IL-18, and tumor necrosis factor (TNF)-α. were significantly higher in PCOS group than the control group (*P* < 0.001). The expressions of HSP70 were significantly lower in serum but higher in ovarian tissues in the PCOS group than the control group. Spearman rank correlation analysis showed strong negative correlation of serum HSP70 levels with T, LH and all the detected inflammatory factors.

**Conclusion:**

The abnormal expression of HSP70 correlated with testosterone and inflammatory factors, which indicates that HSP70 may play an important role in PCOS pathology.

## Background

Polycystic ovary syndrome (PCOS) is one of the most common endocrine disorders in women of reproductive age, estimated prevalence ranges from 6 to 15% [1], but the mechanism of the pathological change is still unclear.

Heat shock proteins (HSPs), known as chaperones, are ubiquitous and highly conserved molecules [2], they belong to a large and diverse group of heat shock-responsive proteins [3]. HSPs can participate in the folding and assembly of intracellular proteins and the degradation of aggregated peptide products then contribute to protein homeostasis [4]. Recent studies showed that HSPs, such as HSP70.2, HSP72 and HSP105/110, can be indicators of stress and efficiency of hormonal action [5].

HSP70 has drawn a lot of attention because of the potent anti-apoptotic, anti- inflammatory, and antioxidant properties [6] and the role of regulation in gametogenesis, pregnancy and so on [7]. Recently, it has been shown that PCOS can cause a systemic chronic nonspecific low-grade inflammation [1]. Elevated serum HSP70 level has been found in brain injury [8] and preeclampsia conditions [9], as well as non-obese PCOS patients [10]. In our previous study, HSP70 was detected in the ovary tissues of rats, which was expressed in granular cells and in the cytoplasm majorly [11].

It’s well known that hyperandrogenism plays a major role in the pathogenesis of PCOS. Dehydroepiandrosterone (DHEA) is the first androgen to appear during female adolescent years and is considered to be the key agent in androgen biosynthesis. There are a lot of studies used DHEA-induced animal model to study the mechanism of PCOS [11–13] since it was first reported by Roy S et al. in 1962 [14].

In the present study, we used DHEA-induced rats PCOS model to extended our previous observations and further investigated the correlations between HSP70 and the hormonal and inflammatory factors, to know the possible roles of HSP70 in the pathogenesis of PCOS.

## Methods

### Animals

Twenty female Sprague–Dawley (SD) rats (aged 23 days and weighted 80-90 g) were purchased from the Laboratory Animal Centre of Wuhan University (Wuhan, China). Animals were kept in groups with free access to food and water and a controlled temperature of 22 ± 2 °C with a 12 h light/12 h dark cycle.

Rats were randomly divided into two groups (*n* = 10 per group), PCOS group and control group. The PCOS rat model was established according to the previous studies [11, 15]. Briefly, rats in PCOS group were subcutaneously injected with 6 mg/100 g DHEA (Aladdin Reagent Co., Ltd., Shanghai, China) which was dissolved in olive oil in the neck once a day for 20 consecutive days, the control group were subcutaneously injected with an equivalent solvent.

Procedures involving animals and their care were conducted in conformity with NIH guidelines (NIH Pub. No.85–23, revised 1996) and was approved by Animal Care and Use Committee of the Wuhan University.

### Samples collection

All the rats were anesthetized with isoflurane during the morning fasting state 12 h after the last administration. One side of fresh ovarian tissue was immediately fixed in 4% paraformaldehyde and embedded in paraffin. The other side of ovarian portions was stored in − 80 °C immediately. After the ovaries were taken out, the chest was opened, about 4 mL blood was taken from the heart, and laid statically and put in 4 °C refrigerator overnight, and centrifuged at 2318×g for 20 min, then the serum was stored in − 80 °C.

### Histology

The paraffin embedded ovarian tissues were sliced into 4 μm-thick sections, and mounted onto the poly-L-lysinecoated slide, some were stained by hematoxylin and eosin (H&E) according to standard procedures (G1005, Servicebio, Wuhan, China), some were for immunohistochemical (IHC) test according to standard procedure as reagent kit (K5007, Agilent Dako, USA). Briefly, deparaffinized sections were incubated in a sodium citrate buffer (pH 6.0), 10 Mm at boiling temperature for 30 min, blocked in 2% PBS-BSA for 20 min, incubated with rabbit anti-rat HSP70 antibody (ab181606, Abcam,UK) at 4 °C overnight, and the slides were subsequently incubated with labeled dextran polymers goat against rabbit IgG at room temperature for 30 min and staining with 3,3′- diamino-benzidine (DAB) for 3 min of twice and and counterstaining with hematoxylin. For negative control PBS was used as a substitute for the primary antibody. IPP6.0 software was used to analyze the optical density of IHC photographs. Three 400 × photographs of each slide were selected for optical density analysis.

### Measurement of serum HSP70, hormone and inflammatory factors levels

Enzyme-linked immunosorbent assay (ELISA) kits were used to measure the serum concentrations of HSP70 (DYC1663E, Minneapolis, MN, USA), follicle stimulating hormone (FSH)(CSB-E06869r, Cusabio Biotech CO.,Ltd., Wuhan, China), luteinizing hormone (LH) (CSB-E12654r, Cusabio Biotech CO.,Ltd., Wuhan, China), estradiol (E2) (CSB-E05110r, Cusabio Biotech CO.,Ltd., Wuhan, China), progesterone (P) (CSB-E07282r, Cusabio Biotech CO.,Ltd., Wuhan, China), testosterone (T)(E-EL-0072c, Elabscience Biotechnology Co.,Ltd., Wuhan, China), insulin (CSB-E05070r, Elabscience Biotechnology Co.,Ltd., Wuhan, China), C-reactive protein (CRP) (E-EL-R0022c, Elabscience Biotechnology Co.,Ltd., Wuhan, China), interleukin (IL)-6(E-EL-R0015c, Elabscience Biotechnology Co.,Ltd., Wuhan, China), IL-18(E-EL-R0567c, Elabscience Biotechnology Co.,Ltd., Wuhan, China), tumor necrosis factor (TNF)-α. (E-EL-R0019c, Elabscience Biotechnology Co.,Ltd., Wuhan, China). There were standard curves used to calculate for all these kits. The coefficients of variation within and between plates were less than 10%.

### Statistical analysis

Data was analyzed using SPSS version 16.0. The Mann–Whitney U test was used to compare the differences between the groups for these abnormal distribution data, and T test was used for these normal distribution data. Regression assessment was made using the Spearman’s rank correlation analysis. A *P* value below 0.05 was considered to be statistically significant.

## Results

### Pathological observations

**Fig. 1:**
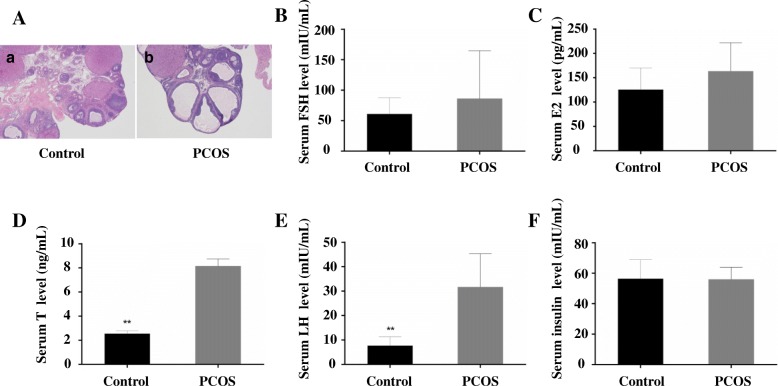
Pathological images and serum concentrations of hormones in PCOS rats. **a**: histopathological images of the ovarian sections of the control (**a**) and the PCOS (**b**) groups (4× magnification, H&E); **b**: Serum FSH levels; **c**: Serum E_2_ levels; **d**: Serum testosterone levels; **e**: Serum LH levels; **f**: Serum insulin levels. *N* = 10, **P < 0.01

Morphological observation showed that the ovarian morphology was normal and there were all types of follicles at different stages include corpus luteum which is an indicator of ovulation. However, the ovaries in PCOS group showed a large number of cystic dilatation and atresia follicles, granular cell layers were reduced to 2–3 layers or even less, theca cell layers were thick and the follicular membrane cells were proliferated (Fig. 1a). The counts of primordial follicles, secondary follicle, preovulatory follicles and corpus luteum were significantly decreased in the PCOS group compared to the control group (*P* < 0.05). Number of cystic and atretic follicles were elevated in the PCOS group compared to the control group (*P* < 0.001) (Table 1). The thickness of granular cell layer was significantly less in the PCOS group compared to the control group (*P* = 0.002).

**Table 1:** Number of ovarian follicles and the thickness of granular cell layer in PCOS and control group (Mean ± SD)

Parameters	PCOS (*n* = 10)	Control (*n* = 10)	*Z* (or *t*)	*P*
Primordial follicle	6.9 ± 3.2	14.6 ± 9.7	−2.767	0.004
Primary follicle	6.4 ± 2.2	11.1 ± 6.8	2.081	0.052
Secondary follicle	6.5 ± 1.3	9.7 ± 4.3	2.234	0.038
Preovulatory follicle	0.4 ± 2.8	0.5 ± 1.0	−3.723	0.000
Cystic follicle	4.5 ± 1.1	1.5 ± 1.0	−3.684	0.000
Corpus luteum	1.6 ± 1.5	5.3 ± 1.3	−3.606	0.000
Atretic follicle	4.7 ± 1.5	0.8 ± 0.8	−3.747	0.000
The thickness of granular cell layer (μm)	0.0698 ± 0.0317	1.3515 ± 0.0462	3.690	0.002

### Serum hormones

Serum concentrations of T and LH were significantly higher in the PCOS group than the control group (*n* = 10, for the comparison of T, *Z* = -3.782, *P* < 0.001; for the comparison of LH, *Z* = -3.630, *P* < 0.001) (Fig. 1d,e), however, the concentrations of E_2_, FSH and insulin didn’t show significant difference between the two groups (*n* = 10, for the comparison of E_2_, *t* = − 1.639, *P* = 0.119; for the comparison of FSH, *t* = − 0.960, *P* = 0.350; for the comparison of insulin, *t* = 0.095, *P* = 0.926) (Fig. 1b, c, f).

### The inflammatory factors

**Fig. 2:**
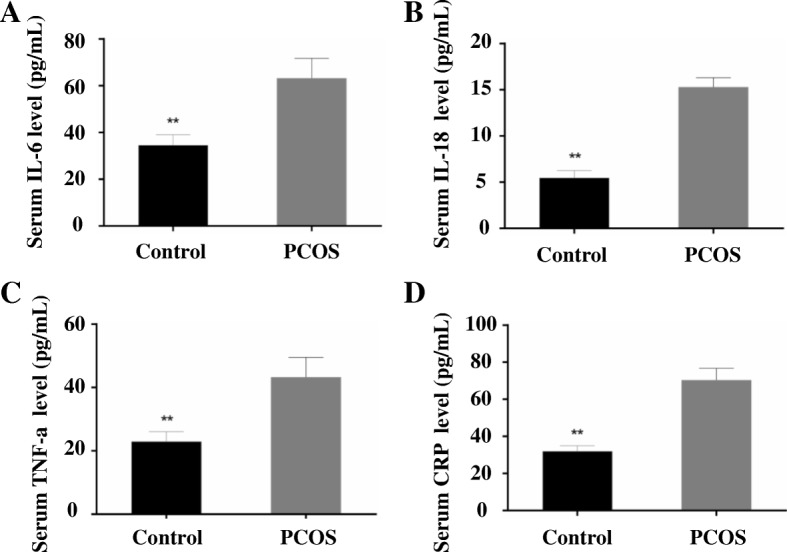
The serum concentrations of inflammatory factors. **a**: Serum IL-6 levels; **b**: Serum IL-18 levels; **c**: Serum TNF-α levels; **d**: Serum CRP levels. *N* = 10, **P < 0.001

All the concentrations of inflammatory factors including IL-6, IL-18, TNF-α and CRP were significantly higher in PCOS group than the control group (*n* = 10, for IL-6, *Z* = -3.790; for IL-18, *Z* = -3.788; for TNF-α, *Z* = -3.790; for the comparison of CRP, *Z* = -3.781; for all of these, *P* < 0.001) (Fig. 2).

### HSP 70 in serum and in ovary

**Fig. 3:**
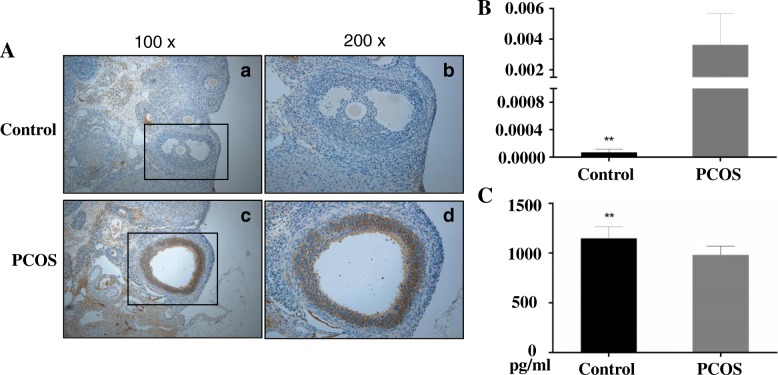
The expression of HSP70 in ovarian tissues and serum. **a**: The IHC images of HSP70 expression in ovarian tissues, **a** and **b** were control group, c and d were PCOS group; **b**: Optical density of IHC slides; **c**: Serum HSP70 levels. *N* = 10, **P < 0.001

The IHC showed that HSP70 was significantly higher in ovarian tissues of the PCOS group than the control group (*n* = 10, *Z* = -3.679, *P* < 0.001) (Fig. 3a and b), however, the concentration of HSP70 in serum was significantly lower in PCOS group than the control group(*n* = 10, *t* = 3.599, *P* = 0.002) (Fig. 3c).

### The correlation of serum HSP70 with hormones and inflammation factors

The correlations between serum HSP70 and hormones as well as inflammatory factors were also investigated (Table 2). Spearman rank correlation analysis showed strong negative correlation of HSP70 with T, LH and all the detected inflammatory factors.

**Table 2:** Spearman rank analysis for the correlation of HSP70, hormones and inflammation factors

		HSP70	T	E_2_	P	FSH	LH	INS	IL-6	TNF-α	IL-18
T	*r*	−.635^**^									
	*P*	**.003**									
E_2_	*r*	−.438	.325								
	*P*	.053	.162								
P	*r*	−.124	.164	.178							
	*P*	.601	.488	.454							
FSH	*r*	−.165	.202	.290	.944^**^						
	*P*	.487	.394	.215	**.000**						
LH	*r*	−.594^**^	.790^**^	.263	−.185	−.131					
	*P*	**.006**	**.000**	.263	.434	.581					
INS	*r*	−.082	.019	.110	−.340	−.256	.001				
	*P*	.731	.937	.645	.143	.275	.998				
IL-6	*r*	−.613^**^	.876^**^	.459^*^	.276	.253	.648^**^	−.060			
	*P*	**.004**	**.000**	**.042**	.239	.282	**.002**	.802			
TNF-α	*r*	−.512^*^	.874^**^	.375	.250	.219	.656^**^	−.116	.948^**^		
	*P*	**.021**	**.000**	.104	.289	.354	**.002**	.627	**.000**		
IL-18	*r*	−.559^*^	.976^**^	.305	.179	.204	.794^**^	−.035	.909^**^	.909^**^	
	*P*	**.010**	**.000**	.191	.450	.389	**.000**	.885	**.000**	**.000**	
CRP	*r*	−.670^**^	.963^**^	.415	.137	.187	.855^**^	.001	.854^**^	.836^**^	.960^**^
	*P*	**.001**	**.000**	.069	.566	.429	**.000**	.998	**.000**	**.000**	**.000**

*P < 0.05, ** *P*<0.01

## Discussion

HSPs are a highly conserved, ubiquitously expressed family and known as molecular chaperones that assist to correct non-covalent assembly of other structures contained polypeptide [16]. HSPs participate in ovarian physiology both in the proliferation/apoptotic mechanisms and the action of the steroidal hormones, and these two key processes are very important in the whole ovarian physiology [17]. As reported by Sirotkin AV et al. [5], there are closely relationships between HSPs and reproductive hormones, HSPs can be produced in follicular granulosa cells and be indicators of stress and efficiency of hormones action, on the other hand, hormones can prevent stress-related changes in HSPs.

HSP70 is one of the members of HSPs and has the functions of potent antioxidant, anti-inflammatory, and anti-apoptotic properties [6]. As reported by previous studies, elevated serum HSP70 is not only involved in the pathogenesis of insulin-resistant disorders, but also in ovarian stress response such as heat shock and malnutrition/serum deprivation in porcine ovarian cells culture [18]. As well as the denote ovarian damage [19], these heat stress could impair estradiol biosynthesis in granulosa cells via increased HSP70 [20], and HSP70 induction in the ovary increases susceptibility to premature ovarian failure due to downregulation of autophagy [7]. In the present study, the serum estradiol levels showed no significant difference between PCOS and control groups, this may due to the high level of testosterone, the precursors of estradiol, can compensate the impaired estradiol biosynthesis by the higher expression of HSP70 in PCOS ovaries.

The present study showed that HSP70 was significantly higher in ovarian tissues in PCOS rats than the controls, but significantly lower in serum. The elevated HSP70 in ovaries might contribute to apoptosis within the ovarian follicle. Velázquez et al. [21] found that HSP70 immunoreactivity was intense in granulosa and theca cells of cystic follicles of PCOS rats, and they inferred the increased HSP70 in the ovary may decrease apoptosis in follicular cysts and delayed regression of these follicles can result a PCO state. Salvetti et al. [22] also found an intense HSP70 immunostaining in the cells of cystic follicles and it associated with the changes in the production of receptors for steroid hormones in ovarian cells. Gao et al. [10] reported that non-obese PCOS women had higher HSP70 concentrations than the control women, which was contrast to the current study.

The regulation of HSP70 expression is mediated by heat shock transcription factor (HSF)-1. Liu et al. [23] reported physiological testosterone can enhance HSP70 expression induced by ischemic preconditioning, which may due to testosterone, can stimulate HSF1 phosphorylation and up-regulate HSP70 mRNA expression, this indicates that testosterone is required for the enhanced expression of HSP70. Kohno et al. [24] reported transiently supra physiologyical testosterone could down-regulate the HSP72 (another member of HSP70 family) expression at a transcriptional level when singly applied, testosterone could induce the suppression of either HSP72 expression or HSF1 activation, which may directly block the trimerization and phosphorylation of HSF1, or indirectly inhibit the HSF1 activation. In the current study, the PCOS rats showed a higher serum concentration of testosterone than controls which is similar to the characteristic of hyperandrogenemia, supra physiological testosterone, of PCOS patients, the spearman rank correlation analysis showed negative strong correlation of serum HSP70 with testosterone (*P* = 0.003) which agrees with the previous studies mentioned above.

The concentrations of serum IL-6, IL-18, TNF-α and CRP were significantly higher in PCOS rats than controls in this study, which is in accordance with previous studies and further confirms the views that there is chronic low-grade inflammation underpinning the pathogenesis of PCOS [10]. The spearman rank correlation analysis showed strong positive correlation of testosterone with all these four inflammatory factors. Hyperandrogenemia is capable of activating mononuclear cells and induce inflammation, which can conversely increase androgen production from ovaries in PCOS [25]. IL-6 is associated with insulin resistance and hyperandrogenism, and it is regarded as an early low-grade chronic inflammatory marker in PCOS patients [26, 27]. Higher TNF-α has a positive correlation with serum androgen level in overweight and obese adolescents PCOS [28], it can cause proliferation of theca-interstitial cells in ovaries and consequently hyperandrogenemia [29]. A hyperplastic theca cell layer and thinner granulosa cell sheet were found in PCOS mice which is consistent with our finding [30]. So these elevated cytokines associated with hyperandrogenemia can impair follicular growth and maturation in PCOS [26].

The spearman rank correlation analysis showed strong negative correlation of HSP70 with all the detected inflammatory factors, but it’s different with previous studies which showed positive correlations between HSP70 and IL-6, TNF-α, TNFRI, IL-1, IL-12 in different pathological conditions such as brain injury [8] and preeclampsia [9]. The possible reason may be that HSP70 is up-regulated in inflamed tissue such as ovaries and may constitute a reliable sensor system for the inflammatory state, but the supra physiological testosterone in serum can suppress the activation of HSF1 and reduce the concentration of HSP70 in serum in PCOS condition.

## Conclusions

In conclusion, HSP70 was significantly higher in ovarian tissues in PCOS rats than the controls, but significantly lower in serum, the abnormal expression of HSP70 correlated with testosterone and inflammatory factors, which indicate that HSP70 may play an important role in PCOS pathology.

## Abbreviations

CRP: C-reactive protein
DHEA: Dehydro-epiandrosterone
E2: Estradiol
FSH: Follicle stimulating hormone
HSP70: Heat shock protein
IL-18: Interleukin 18
IL-6: Interleukin 6
LH: Luteinizing hormone
PCOS: Polycystic ovary syndrome
T: Testosterone
TNF-α: Tumor necrosis factor-α
